# Diagnostic performances of Claude 3 Opus and Claude 3.5 Sonnet from patient history and key images in Radiology’s “Diagnosis Please” cases

**DOI:** 10.1007/s11604-024-01634-z

**Published:** 2024-08-03

**Authors:** Ryo Kurokawa, Yuji Ohizumi, Jun Kanzawa, Mariko Kurokawa, Yuki Sonoda, Yuta Nakamura, Takao Kiguchi, Wataru Gonoi, Osamu Abe

**Affiliations:** 1 Department of Radiology, Graduate School of Medicine, The University of Tokyo, 7-3-1 Hongo, Bunkyo-ku, Tokyo 113-8655 Japan; 2 Department of Radiology, Ichinomiyanishi Hospital, 1 Hira Kaimei, Ichinomiya-shi, Aichi 494-0001 Japan

**Keywords:** Large language model, Artificial intelligence, Claude 3 Opus, Claude 3.5 Sonnet, ChatGPT

## Abstract

**Purpose:**

The diagnostic performance of large language artificial intelligence (AI) models when utilizing radiological images has yet to be investigated. We employed Claude 3 Opus (released on March 4, 2024) and Claude 3.5 Sonnet (released on June 21, 2024) to investigate their diagnostic performances in response to the Radiology’s Diagnosis Please quiz questions.

**Materials and methods:**

In this study, the AI models were tasked with listing the primary diagnosis and two differential diagnoses for 322 quiz questions from Radiology’s “Diagnosis Please” cases, which included cases 1 to 322, published from 1998 to 2023. The analyses were performed under the following conditions: (1) Condition 1: submitter-provided clinical history (text) alone. (2) Condition 2: submitter-provided clinical history and imaging findings (text). (3) Condition 3: clinical history (text) and key images (PNG file). We applied McNemar’s test to evaluate differences in the correct response rates for the overall accuracy under Conditions 1, 2, and 3 for each model and between the models.

**Results:**

The correct diagnosis rates were 58/322 (18.0%) and 69/322 (21.4%), 201/322 (62.4%) and 209/322 (64.9%), and 80/322 (24.8%) and 97/322 (30.1%) for Conditions 1, 2, and 3 for Claude 3 Opus and Claude 3.5 Sonnet, respectively. The models provided the correct answer as a differential diagnosis in up to 26/322 (8.1%) for Opus and 23/322 (7.1%) for Sonnet. Statistically significant differences were observed in the correct response rates among all combinations of Conditions 1, 2, and 3 for each model (*p* < 0.01). Claude 3.5 Sonnet outperformed in all conditions, but a statistically significant difference was observed only in the comparison for Condition 3 (30.1% vs. 24.8%, *p* = 0.028).

**Conclusion:**

Two AI models demonstrated a significantly improved diagnostic performance when inputting both key images and clinical history. The models’ ability to identify important differential diagnoses under these conditions was also confirmed.

## Introduction

Large language artificial intelligence (AI) models, which are designed to understand and generate human-like text based on an input, have recently demonstrated remarkable capabilities across various domains [1]. For instance, in the field of diagnostic imaging, Ueda et al. [2] conducted a study using ChatGPT’s GPT-4 model [3] and found it to correctly answer 54% (170 of 313) of Radiology’s Diagnoses Please quiz questions based solely on textual information from clinical history and imaging findings. Horiuchi et al.[4] also demonstrated that ChatGPT’s GPT-4 model achieved a diagnostic accuracy rate of 50% (50/100 cases) in American Journal of Neuroradiology’s “Case of the Week” cases based on textual information from clinical history and imaging findings. These findings highlight the potential use of AI in diagnostic imaging. However, at present, the diagnostic accuracy of the AI models using radiological images remains unclear. Furthermore, the ability to output a series of highly probable differential diagnoses and their respective accuracies, which are crucial in daily clinical practice, has not been previously investigated using AI models. Claude 3 Opus and Claude 3.5 Sonnet [5], large language AI models developed by Anthropic (California, United States), can read and analyze not only input text but also image data.

In this study, Claude 3 Opus (released March 4, 2024) and the latest model Claude 3.5 Sonnet (released June 21, 2024) were used to examine its diagnostic performance in relation to the Radiology’s “Diagnosis Please” quiz under three input conditions: (1) clinical history alone, (2) clinical history and imaging findings, and (3) clinical history and key images. In addition, the diagnostic performances of the models when instructed to list differential diagnoses were also evaluated and compared.

## Materials and methods

**Fig. 1:**
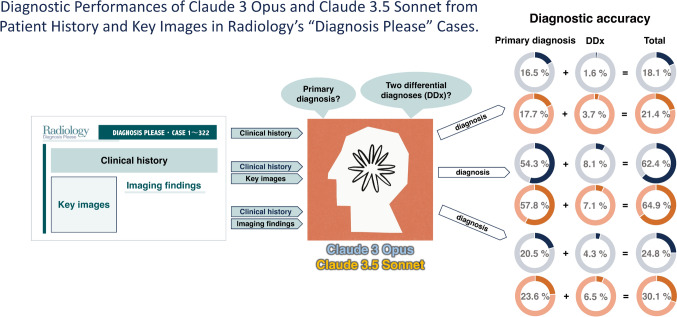
Overview of this study

An overview of this study is presented in Fig. 1. We used Claude 3 Opus (Anthropic, California, United States) (released on March 4, 2024, accessed on June 27, 2024) (https://claude.ai/) and Claude 3.5 Sonnet (Anthropic, California, United States) (released on June 21, 2024, accessed on June 27, 2024) to list the primary diagnosis and two differential diagnoses for 322 quiz questions from Radiology Diagnosis (https://dxp.rsna.org/), spanning cases 1 to 322, published between 1998 and 2023. The analyses were conducted under the following three input data conditions:

1. Condition 1: submitter-provided clinical history (text) alone
2. Condition 2: submitter-provided clinical history and imaging findings (text)
3. Condition 3: clinical history (text) and key images (PNG file)

Application programming interfaces were used to access each model (Claude 3 Opus: claude-3-opus-20240229 and Claude 3.5 Sonnet: claude-3-5-sonnet-20240620) on June 27, 2024. To ensure reproducibility, we specified the generation parameters for all models as temperature = 0.0 and top-*p* = 1.0. To prevent previous inputs from influencing subsequent ones, inputs were conducted in an independent session for each case for each condition. The prompt was as follows [4]: “As a physician, I plan to utilize you for research purposes. Assuming you are a hypothetical physician, please walk me through the process from differential diagnosis to the most likely diagnosis and the next two most likely differential diagnoses step-by-step based on the attached patient’s information.” Each prompt was submitted to the models only once, and the first response generated was used for evaluation. When extracting the submitter-identified imaging findings, two trainee radiologists and one board-certified diagnostic radiologist with 11 years of experience meticulously removed sentences containing answers to ensure analysis integrity. The accuracy of the primary diagnosis and two differential diagnoses generated by the models were determined by consensus between three board-certified diagnostic radiologists with 8, 11, and 19 years of experience. Ethical approval was not required as this study exclusively used published articles. McNemar’s test was used to assess the difference in correct response rates for the overall accuracy under Conditions 1, 2, and 3 for each model and between the models. Two-sided *p*-values < 0.05 were considered statistically significant. Statistical analyses were performed using R (version 4.1.1; R Foundation for Statistical Computing, Vienna, Austria).

## Results

The results are summarized in Table 1 (Table 1). Both models showed diagnostic performance in the order of Condition 2 > Condition 3 > Condition 1 (all ps < 0.01). In comparing the diagnostic performance between models, Claude 3.5 Sonnet outperformed in all conditions, but a statistically significant difference was observed only in the comparison for Condition 3 (30.1% in Sonnet vs. 24.8% in Opus, *p* = 0.028).

**Table 1:** Diagnostic performance

Model	Input data	Correct primary diagnosis	Correct differential diagnosis	Overall accuracy
Claude 3 Opus	History alone	53/322 (16.5%)	5/322 (1.6%)	58/322 (18.0%)
	History plus imaging findings	175/322 (54.3%)	26/322 (8.1%)	201/322 (62.4%)
	History plus key images	66/322 (20.5%)	14/322 (4.3%)	80/322 (24.8%)
Claude 3.5 Sonnet	History alone	57/322 (17.7%)	12/322 (3.7%)	69/322 (21.4%)
	History plus imaging findings	186/322 (57.8%)	23/322 (7.1%)	209/322 (64.9%)
	History plus key images	76/322 (23.6%)	21/322 (6.5%)	97/322 (30.1%)

## Discussion

In this study, we evaluated the diagnostic performances of Claude 3 Opus and Claude 3.5 Sonnet, large language AI models, in determining radiology diagnoses and answering questions based on three types of input information: (i) clinical history alone, (ii) clinical history and imaging findings, and (iii) clinical history and key images.

Ueda et al. [2] demonstrated that, using the ChatGPT’s GPT-4 model, a large language AI model, with text information from clinical history and imaging findings as input, ChatGPT correctly answered 54% of questions. Similarly, Li et al. [6] showed a 17% improvement in diagnostic accuracy when using the GPT-4 model compared with the GPT-3.5 model of ChatGPT when inputting text information from clinical history and imaging findings. Large language AI models continue to evolve, enabling users to read image information. However, the diagnostic performance of AI models that use radiological images in conjunction with textual information has not yet been investigated.

According to the results, Claude 3 Opus and Claude 3.5 Sonnet provided an accurate diagnosis in 24.8% (80/322) and 30.1% (97/322) of cases, respectively, when inputting key images and clinical history. These showed a 6.8% (Opus) and 8.7% (Sonnet) increases with statistically significant differences in accuracy compared with providing clinical history alone, potentially reflecting the imaging diagnostic performance of AI models. However, these were inferior to the accuracy achieved using clinical history and submitter-provided imaging findings (Opus: 24.8% vs. 62.4%; Sonnet: 30.1% vs. 64.9%; with significant differences). The 54.3% (175/322) and 57.8% (186/322) accuracy rates of the primary diagnoses obtained by Claude 3 Opus and Claude 3.5 Sonnet when using clinical history and imaging findings were nearly equivalent to the 54% shown by Ueda et al. [2] using the GPT-4 model.

Owing to real-world complexities, there may be more than one possible diagnosis in daily clinical practice, necessitating the reporting of potential differential diagnoses. In this study, the AI models presented two differential diagnoses, in addition to the primary diagnosis. They succeeded in presenting the correct answer as a differential diagnosis in up to 8.1% (26/322, Opus) and 7.1% (23/322, Sonnet) of cases, resulting in an accuracy of up to 62.4% (201/322, Opus) and 64.9% (209/322, Sonnet) when the clinical history and imaging findings were input.

The results of this study suggest that large language AI models, in their current state, may be more effectively utilized as support tools by radiologists when formulating differential diagnoses based on accurate image interpretation and clinical history rather than as standalone solutions for replacing radiologists in the image interpretation process. Although these AI models demonstrate a certain level of usefulness in assisting radiologists, their ability to interpret image findings solely from the images themselves and provide meaningful contributions to diagnosis requires improvement and is still in the early stages of development. In this study, Claude 3.5 Sonnet performed better in all conditions compared to Claude 3 Opus, although the difference reached the significance in only the comparison under Condition 3. As the performance of large language AI models advances and innovations in the provided information emerge, there is potential for further improvements in their diagnostic capabilities. To fully realize this potential, additional research and validation are needed to explore the evolving role of AI models in the field of diagnostic imaging, including a prospective study to investigate the diagnostic performance by letting the models to solve the quiz cases with no answer explanations yet published.

## Abbreviation

AI: Artificial intelligence
